# Unique Pulmonary Hypertension in Young Children: A Case Series Study

**DOI:** 10.3390/children9071064

**Published:** 2022-07-17

**Authors:** I-Chen Chen, Hsiu-Lin Chen, Yi-Ching Liu, Yen-Hsien Wu, Shih-Hsing Lo, Jong-Hau Hsu, Hsin-Ling Yin, Jui-Sheng Hsu, Bin-Nan Wu, Zen-Kong Dai

**Affiliations:** 1 Department of Pediatrics, Kaohsiung Medical University Hospital, Kaohsiung 80756, Taiwan; yljane@gap.kmu.edu.tw (I.-C.C.); chenhl.chency108@gmail.com (H.-L.C.); furtherchia@gmail.com (Y.-C.L.); eddiewu1986@gmail.com (Y.-H.W.); allenjay66@gmail.com (S.-H.L.); jhh936@yahoo.com.tw (J.-H.H.); 2 Department of Pediatrics, School of Medicine, College of Medicine, Kaohsiung Medical University, Kaohsiung 80756, Taiwan; 3 Graduate Institute of Medicine, College of Medicine, Kaohsiung Medical University, Kaohsiung 80756, Taiwan; e3124@ms16.hinet.net; 4 Department of Respiratory Therapy, College of Medicine, Kaohsiung Medical University, Kaohsiung 80756, Taiwan; 5 Department of Clinical Forensic Medicine, Kaohsiung Medical University Hospital, College of Medicine, Kaohsiung Medical University, Kaohsiung 80756, Taiwan; schoolyin@gmail.com; 6 Department of Radiology, Kaohsiung Medical University, Kaohsiung 80756, Taiwan; 7 Department of Pharmacology, School of Medicine, College of Medicine, Kaohsiung Medical University, Kaohsiung 80756, Taiwan

**Keywords:** pulmonary hypertension, children, bronchopulmonary dysplasia, Swyer–James–Macleod syndrome, taurine, renin

## Abstract

Pediatric pulmonary hypertension (PH) has a similar clinical presentation to the adult disease but is associated with several additional disorders and challenges that require a specific approach for their fulminant course. With improved care for premature infants, various forms of pulmonary vascular disease have been found in children that did not previously exist. Pediatric PH can begin in utero, resulting in pulmonary vascularity growth abnormalities that may persist into adulthood. Here, we retrospectively reviewed several unique pediatric PH cases from 2000 to 2020 at Kaohsiung Medical University Hospital, Taiwan, a tertiary teaching hospital. Their comorbidities varied and included surfactant dysfunction, bronchopulmonary dysplasia, premature closure of the ductus arteriosus, high levels of renin and aldosterone, and Swyer–James–Macleod syndrome. Their clinical profiles, radiological characteristics, echocardiography, pulmonary angiogram, and therapeutic regimens were recorded. Further, because the underlying causes of pediatric PH were complex and markedly different according to age, adult PH classification may not be applicable to pediatric PH in all settings. We also classified these cases using different systems, including the Panama classification and the Sixth World Symposium on PH, and compared their advantages and disadvantages.

## 1. Introduction

The classification of pulmonary hypertension (PH) has undergone a series of changes since the first World Symposium on Pulmonary Hypertension (WSPH), held in 1973. The latest definition of PH was published during the sixth WSPH, which was held in 2018 in Nice, France, and proposed revising the hemodynamic definition of PH, lowering its threshold from ≥25 to >20 mmHg, and classifying the etiology of PH into the following five groups: (1) pulmonary arterial hypertension, (2) PH due to left heart disease, (3) PH due to lung disease or hypoxia, (4) PH due to pulmonary artery (PA) obstructions, and (5) PH with unclear or multifactorial mechanisms [1].

The definition and clinical presentation of PH in children are the same as those in adults; however, they do not carry any implication regarding the presence or absence of pulmonary vascular disease and the precapillary and postcapillary forms [2]. Several important aspects of pediatric PH are not included in the classification, for example, the fetal origins of pulmonary vascular disease; an uncertain approach to pulmonary vascular disease; the developmental mechanisms of children being different from those of adults; the perinatal maladaptation, maldevelopment, and pulmonary vascular hypoplasia; and adult survivors of pediatric pulmonary vascular disease [3].

The Pulmonary Vascular Research Institute meeting, held in Panama in 2011, developed a new classification for pediatric pulmonary hypertensive vascular disease, focusing on PH in childhood [4,5]. Critically, it allowed the identification of more specific diseases within pediatric PH vascular disease. It outlined pediatric PH vascular disease diagnostic classification, including 10 main categories and 109 subcategories, which testifies to the complexity of PH pathophysiology in newborns and children’s growth and development [4]. The 10 main categories are as follows:

1. Prenatal or developmental pulmonary vascular hypertensive disease;
2. Perinatal pulmonary vascular maladaptation;
3. Pediatric cardiovascular disease;
4. Bronchopulmonary dysplasia (BPD);
5. Isolated pediatric pulmonary vascular hypertensive disease;
6. Multifactorial pediatric pulmonary vascular hypertensive disease associated with other birth defects;
7. Pediatric lung disease;
8. Pediatric thromboembolic disease;
9. Pediatric hypobaric hypoxic exposure;
10. Pediatric pulmonary hypertensive vascular disease associated with other systemic disorders.

The unique, distinctive features of pediatric PH were recognized during the fifth WSPH and confirmed in the sixth. Looking for uniformity, an attempt was made to adapt the adult classification to pediatric patients. However, all of these commendable classifications are very complex and may not permit quick comprehension by clinicians. Calcaterra et al. proposed a simplified classification method, consisting of only five groups: (1) neonatal (neonatologist), (2) cardiac (pediatric cardiologist), (3) developmental (general practitioner, pediatrician, or pulmonologist), (4) idiopathic (general practitioner, pediatrician, or pulmonologist), and (5) syndromic PH (pediatrician) [2]. This approach is mainly based on the kind of specialized physician who first faces and provides care for a child with suspected PH [2].

Due to the disparities between pediatric and adult PH and the complexity of its development, in this study, we share our experiences in confronting and managing unique PH in young children. We classify these cases using these different classification systems and compare their advantages and disadvantages.

## 2. Materials and Methods

This retrospective and descriptive study included patients with a diagnosis of PH who were followed up at the Pediatric Cardiopulmonary Department of Kaohsiung Medical University Hospital, Taiwan. The protocol was approved by the Institutional Ethics Research Committee of that institution (KMUHIRB-SV(I)-20220026). The medical records of the enrolled children were reviewed retrospectively by the staff of that institution from January 2000 to December 2021. A diagnosis of PH was based on clinical presentation and echocardiography. Chest radiography (CXR), cardiac catheterization, high-resolution computed tomography of the chest, or pulmonary ventilation and perfusion scintigraphy (a V/Q scan) may have been arranged, allowing a survey of the diverse etiology of PH. Demographic information, including age and sex, clinical presentations of disease onset, pharmacological treatment, and echocardiography and radiology reports were obtained. We also sought to classify these cases using these different classification systems, including 6th WSPH, Panama categories, and a simplified classification method.

## 3. Results

### 3.1. Case 1

**Figure 1:**
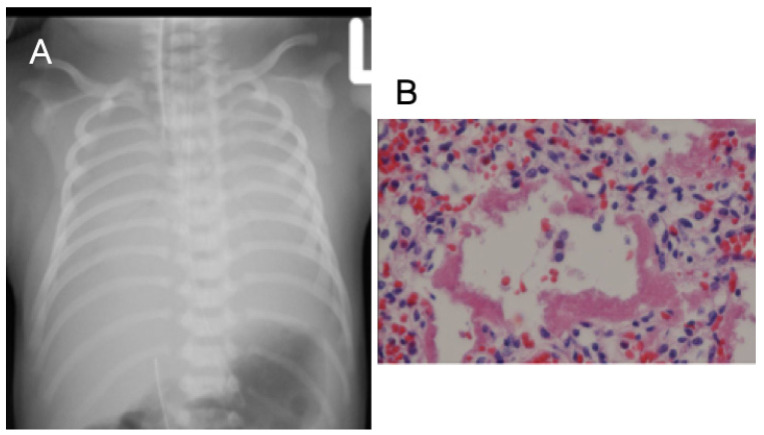
(**A**) Chest radiography of a male neonate presenting with cyanosis and pulmonary hypertension, revealing diffuse ground glass lungs with low volumes; (**B**) lung section showing that the alveolar ducts, alveoli, and terminal and respiratory bronchioles are lined with an eosinophilic hyaline membrane with necrotic alveolar lining cells and fibrin (40×).

A male infant was born with a gestational age of 36 + 5 weeks, birth body weight (BBW) of 2860 g, elective cesarean section, and Apgar scores of 8 and 9 at 1 and 5 min, respectively. He was born to a 40-year-old Taiwanese mother (G1P1A0), who had regular prenatal examinations and no abnormality was reported except that the baby’s chromosome report showed 47, XXY. The delivery course was smooth but general cyanosis was observed at birth. The baby’s SpO_2_ was approximately 70% with a 5 L/min O_2_ supply, followed by bradycardia. He was intubated immediately and transferred to our neonatal intensive care unit for further care. His first CXR showed bilateral white-out (Figure 1A). Transthoracic echocardiography (TTE) demonstrated a dilated right ventricle (RV) and main PA, and a right-to-left shunt through a patent ductus arteriosus (PDA) and a patent foramen ovale (PFO) with an estimated systolic pulmonary artery pressure (SPAP) of 70 mmHg. Prostaglandin E1 was administered. However, the desaturation and bradycardia persisted, and the patient died in the sixth hour of life. An autopsy of his lung revealed eosinophilic hyaline membrane lining (Figure 1B), which was compatible with neonatal respiratory distress syndrome. His PH was classified as sixth WSPH group 1, Panama classification group 6, and simplified classification group 1. The classifications for all cases are listed in Table 1.

### 3.2. Case 2

**Figure 2:**
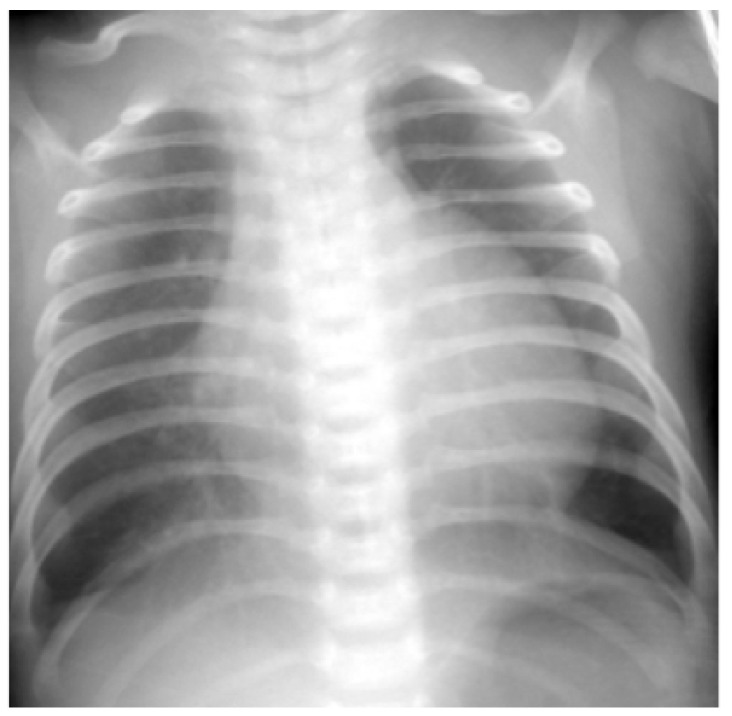
Chest radiography of a 52-day-old full-term male infant presenting with cyanosis and diagnosed with pulmonary hypertension showed bilateral hyperlucent lung and cardiomegaly with a cardiothoracic ratio of 0.64.

A 52-day-old, full-term boy was admitted because of poor feeding, cyanosis, and malnutrition. At admission, he had an SpO_2_ of 65% on ventilatory support. His CXR showed only cardiomegaly without other increased infiltration (Figure 2). TTE demonstrated a severely dilated RV and main PA, D-shaped left ventricle, and a right-to-left shunt through a PDA, with an estimated SPAP of 84 mmHg. She was immediately rescued with venoarterial-mode extracorporeal membrane oxygenation and administered digoxin, furosemide, inhaled nitric oxide (iNO), sildenafil, and inhaled iloprost. Cardiac catheterization demonstrated 119/72 (mean 93) mmHg with an O_2_ saturation of 53% in the PA, and 9 and 78/14 mmHg in the left atrium and left ventricle, respectively. Regarding pulmonary-to-systemic flow, the flow (Qp/Q_S_) and pressure (Ppa/Pao) ratios were 0.9 and 1.5, respectively. A pulmonary vascular resistance of 21.8 units∙m^2^ with a positive acute vasodilator test was also indicated. Based on all of these data points together, idiopathic pulmonary arterial hypertension was diagnosed. The patient expired 1 month after admission. Her PH was classified as sixth WSPH group 1, Panama classification group 2, and simplified classification group 1.

### 3.3. Case 3

**Figure 3:**
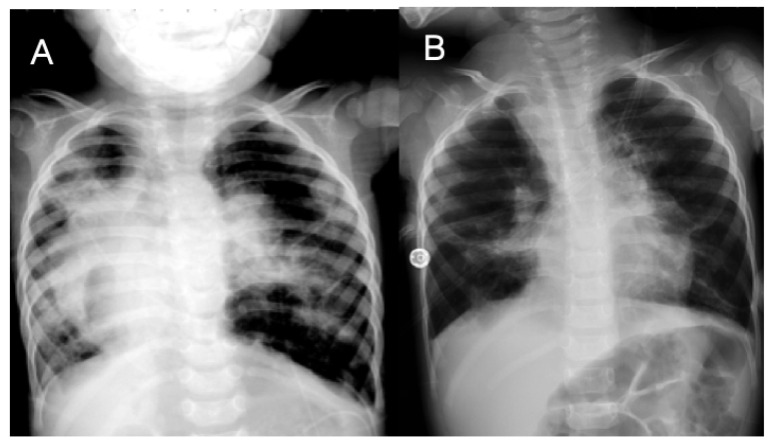
An extremely preterm male infant was diagnosed with bronchopulmonary dysplasia (BPD) and pulmonary hypertension. Chest radiography at the age of 3 years demonstrated dextrocardia, widespread coarse interstitial markings, atelectasis, and regions of hyperexpansion (**A**). A preterm female infant was diagnosed with BPD and pulmonary hypertension. Her chest radiography at the age of 2 years also showed the coarse interstitial markings, atelectasis, and regions of hyperexpansion (**B**) typical of BPD.

A male premature infant, vaginally delivered at 25 weeks of gestation, with a birth weight of 675 g, was rescued with high-frequency oscillatory ventilation (HFOV). He was diagnosed as BPD and PH at 1 year old, presenting with desaturation and dyspnea. His CXR showed dextrocardia, widespread coarse interstitial markings, atelectasis, and regions of hyperinflation (Figure 3A). His TTE revealed a dilated RV with an estimated SPAP of 93 mmHg. Cardiac catheterization indicated 94/71 (mean 78) mmHg with an acute vasoreactive response in the PA, and a Qp/Qs of 1.3, a Pp/Ps of 1.0, and an Rp/Rs of 0.7. After furosemide, sildenafil, and inhaled iloprost were given, his situation was unstable. The patient died of right heart failure. His PH was classified as sixth WSPH group 3, Panama classification group 4, and simplified classification group 1.

### 3.4. Case 4

A female premature infant, born at 27 + 3 weeks of gestation with a birth weight of 1060 g, was diagnosed as BPD associated with O_2_-dependent PH. She had been transferred to our hospital because of a decreased SpO_2_ of 60% and acute respiratory failure. CXR demonstrated heterogenous fibrosis and emphysematous change (Figure 3B). TTE demonstrated an estimated SPAP of 66 mmHg with a bilateral shunt through a PFO. She was treated with iNO and achieved a stable condition after a combination of sildenafil and furosemide was given. She had regular follow-ups until 14 years old. Her PH was classified as sixth WSPH group 3, Panama classification group 4, and simplified classification group 1.

### 3.5. Case 5

**Figure 4:**
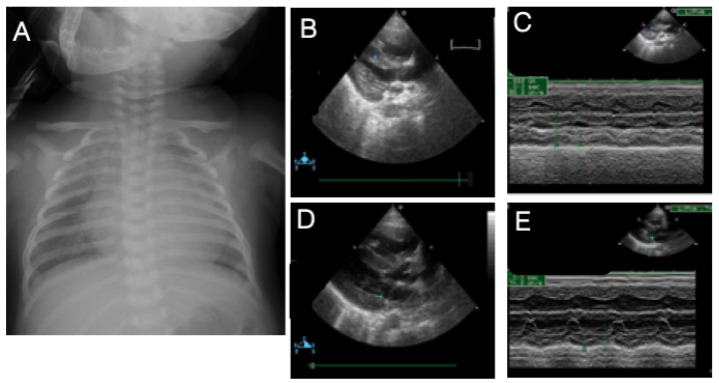
A 3-month-old female infant was diagnosed with pulmonary hypertension with high levels of renin and aldosterone syndrome. Chest radiography showed a mild ground glass appearance and cardiomegaly with a cardiothoracic ratio of 0.75 (**A**). Long-axis echocardiographic images (two-dimensional (**B**) and M-mode (**C**)) demonstrated a hypertrophic right ventricle, which was much improved 6 months after candesartan (angiotensin receptor blocker) and captopril (angiotensin-converting enzyme inhibitor) were given (**D**,**E**).

A 1-day-old female, full-term with a BBW of 3000 g and mega cisterna magna noted prenatally, was intubated immediately because of a delay in initial crying and poor activity at birth. Cardiomegaly and ground glass appearance were noted in CXR (Figure 4A). In addition, diabetes, infantile cerebral palsy, and chronic hydrocephalus were noted. The TTE indicated a large left-to-right shunt via a PDA, atrial septum defect secundum, an increased estimated SPAP of 95 mmHg, and a hypertrophic RV (Figure 4B,C). Her SpO_2_ was noted at approximately 90% on a FiO_2_ of 60%, and she was rescued with HFOV and iNO. Inhaled iloprost and sildenafil were subsequently given. Cardiac catheterization demonstrated a large PDA with a Qp/Qs of 3:1, 91/72 (78) mmHg. An O_2_ of 85.7% was measured in the PA, and 4 mmHg and 54.3% O_2_ were found in the RA. Two weeks after PDA ligation was performed, high levels of renin (95.43 ng/mL/h; normal: 0.84–2.5 ng/mL/h) and aldosterone (83.01 ng/mL/h; normal: 4–31 ng/mL/h) were detected. Therefore, she was placed on candesartan (angiotensin receptor blocker), captopril (angiotensin-converting enzyme inhibitor), furosemide, and sildenafil under a diagnosis of PH associated with high renin levels. After 6 months, the levels of both renin and aldosterone decreased, the estimated SPAP decreased to 43 mmHg, the hypertrophic RV improved (Figure 4D,E), and she began to gradually gain body weight. The patient expired at 7 years old because of sudden cardiac arrest. Her PH was classified as sixth WSPH groups 5, Panama classification group 6, and simplified classification group 1.

### 3.6. Case 6

**Figure 5:**
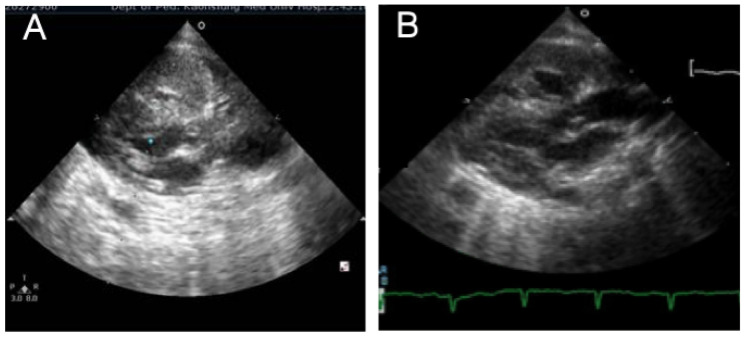
Long-axis echocardiographic image in a 1-day-old female newborn revealed a hypertrophic right ventricle without patent ductus arteriosus noted (**A**), which improved 5 days later (**B**).

The patient was a female neonate, born to a 29-year-old Taiwanese mother (G3P3A0) via Caesarean section, with a BBW of 1815 g. A prenatal cardiac echogram revealed a dilated RV and early closure of the ductus arteriosus. At birth, her SpO_2_ was approximately 70–85% on a FiO_2_ of 60%; she was intubated and given intravenous Prostaglandin E1. Interestingly, the mother had ingested approximately 100 mL per day of Paolyta-B Liq from the third trimester. TTE performed immediately after birth demonstrated marked hypertrophy in the RV and an estimated SPAP of 55 mmHg, no evidence of PDA, and a right-to-left shunt through a PFO (Figure 5A). She was supported by HFOV and iNO because of a decreased SpO_2_ level of 70% until day 4. We successfully weaned her from the ventilator on day 6. She presented with normal oxygen saturation in room air, and adequate feeding and growth. Her PH was not definitively classified under the sixth WSPH system but was Panama classification group 1 and simplified classification group 1.

### 3.7. Case 7

**Figure 6:**
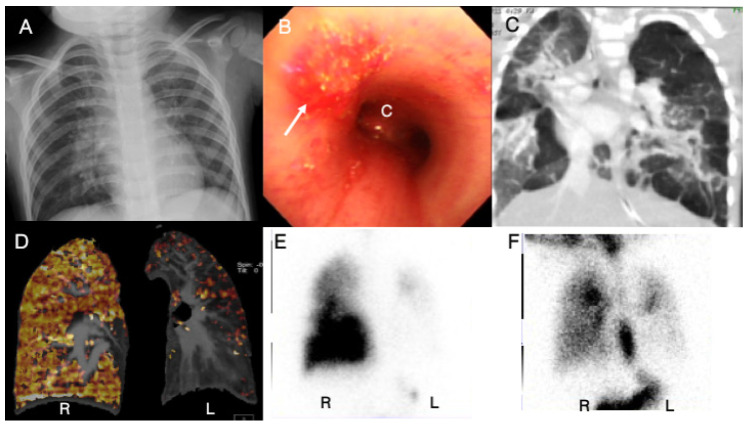
A 3-year-old boy presenting with hemoptysis and dyspnea was diagnosed with Swyer–James–Macleod syndrome and pulmonary hypertension. Chest radiography revealed a unilateral hyperlucent lung (**A**); a bronchoscopy revealed several erosions and bleeding from the trachea (arrow) (**B**); coronal-view high-resolution computed tomography of the chest revealed regional ground-glass opacities and focal emphysema, particularly in the left upper lobe (**C**); dual-energy computer tomography revealed areas of decreased lung attenuation, associated with decreased pulmonary blood vasculature in the left lung (**D**); perfusion scintigraphy revealed marked defects in left lung fields (**E**); and ventilation scintigraphy revealed a marked defect corresponding to the previous lab tests (**F**). C, carina; R, right side; L, left side.

A three-year-old boy was diagnosed with Swyer–James–Macleod syndrome (SJMS) with the presentation of hemoptysis, dyspnea, anemia, and a history of frequent infection in early childhood. His SpO_2_ was 91% without oxygen supply at the initial assessment. An echocardiogram showed a hypoplastic left PA with an estimated SPAP of 59 mmHg. His CXR demonstrated a unilateral hyperlucent lung (Figure 6A). Bronchoscopy revealed erosions and bleeding from the trachea and bilateral bronchus (Figure 6B). High-resolution computed tomography showed focal emphysematous changes and a ground glass appearance (Figure 6C). Dual-energy computer tomography revealed a global perfusion defect in the left lobe (Figure 6D), and a pulmonary V/Q scan revealed a global reduction in perfusion in the left lobe corresponding to that perfusion defect. He was placed on furosemide, captopril, digoxin, and sildenafil. He was moved to another hospital, where he expired at 4 years old. His PH was classified as sixth WSPH group 5, Panama classification group 5, and simplified classification group 3.

## 4. Discussion

Children are not small adults; PH in neonates, infants, children, adolescents, and young adults is a complex condition. Its causes are varied and can be associated with several cardiac, pulmonary, and systemic diseases that contribute to morbidity and mortality. Although the most common PH classification system used, WSPH, was never specifically limited to adult patients, utilizing it for pediatric subjects can be less than ideal. At the 2011 Pulmonary Vascular Research Institute meeting in Panama, a new classification for the pediatric pulmonary hypertensive vascular disease was developed, focused on the etiologies of PH in childhood [4,5]. The WSPH classification does not completely characterize or individualize any subgroup of pediatric PH, whereas the Panama classification provides a system of categories containing the unique aspects of pediatric PH, such as prenatal, developmental, and growing disorders that result in PH in childhood [3]. This is why we shared our experience in handling some unique cases of PH in young children and using different PH classification systems for them.

Our first case had Klinefelter syndrome, which affects males with hypogonadism until adulthood. The syndrome is usually accompanied by metabolic, morphological, and neurobehavioral manifestations, and sometimes by venous thromboembolic diseases [6]. No studies have assessed the association between Klinefelter syndrome and PH, and we may speculate that this PH was independent of the syndrome. Moreover, the autopsy showed a typical pattern of hyaline membrane disease, with neonatal respiratory distress syndrome clinically. The alveolar epithelial cells and bronchiolar epithelium were injured and necrotic. As our case was a near-full-term infant, and his disease progression was so fast that we did not give him surfactant in time, we must strongly suspect surfactant dysfunction.

In our second case, the prenatal history was unremarkable. The patient suffered from progressive dyspnea immediately after birth, without specific comorbidity or reasons associated with PH. In this case, his PH classification may belong to group 1 under the sixth WSPH for idiopathic PH; however, according to the Panama classification, we may assume he is in group 2, perinatal pulmonary vascular maladaptation. There are scant data on how much time is appropriate to adapt from intrauterine to neonatal life, and thus, the debate over the timing of occurrence between persistent pulmonary hypertension in the newborn and late-onset PH has continued. Some researchers hypothesize that the former typically starts within the first days of life, based on the idea that fetal circulation should adapt to extrauterine life within the first day(s) [7]; however, others hypothesize that it occurs throughout the neonatal period [8]. Some infants develop early onset PH due to the unique adaptive capabilities of the immature host, but many do not have clinical evidence of early pulmonary vascular dysfunction. The development of the pulmonary circulation is critical in the development of fetal lungs and continues through infancy and childhood. Any perinatal vascular insults may result in abnormal vascular structure or dysfunction, including decreased angiogenic signaling and vascular endowment, impaired vasoreactivity by increased pulmonary artery endothelial dysfunction and remodeling, or enhanced genetic susceptibility to pulmonary vascular disease through epigenetic modifications [9]. We speculate that the primary perinatal insults in Case 2 induced the maladaptation in the pulmonary vascular system. In fragile infants, the distinction between primary and secondary problems is not straightforward, underlining the need for a consensus definition for clinical practice and research.

BPD is the most common adverse outcome for extremely premature babies and can result in complications that may extend into adulthood [10]. One of the significant contributory factors to morbidity and mortality in infants with BPD is the development of PH [11]. There is no doubt that BPD-PH under the sixth WSPH classification is group 3 and under the Panama classification is group 4. The pathogenesis of BPD developing into PH is multifactorial, and the incidence of PH in infants with BPD ranges from 17% to 37% [12,13,14]. The diagnosis of BPD-related PH depends on echocardiography and is classified as early or late (≤ or >28 days after birth, respectively). BPD with late PH has a mortality rate as high as 36.4% and is associated with a prolonged duration of stay and higher healthcare costs [15]. In our series, we had two cases (Case 3 and Case 4) diagnosed with late PH. They both received furosemide and sildenafil as initial management, with optional inhaled iloprost as a second-line pharmacotherapy for PH in BPD [16]. In the absence of randomized clinical trial data, no conclusion can be reached regarding the use of agents in BPD-PH. Several studies have reported that sildenafil and bosentan are frequently used for the acute and long-term treatment of infants with BPD-PH [17,18,19]. Indeed, the use of medications to target PH in infants is based on expert opinion, experience, and drug availability, and a large-scale prospective cohort study is needed to clarify this issue.

The renin–angiotensin–aldosterone system (RAAS) has been implicated to play a causative role in PH [20,21,22,23]. The RAAS affects the occurrence and development of left ventricular hypertrophy of the heart mainly through cell proliferation, cell hypertrophy, and partial myocardial hypertrophy [24]. The concentration of renin plays a rate-limiting role in the production of angiotensin I, while angiotensinogen plays an important regulatory role in the production of angiotensin II [25]. It is reported that increased renin–angiotensin activity and elevated levels of aldosterone were noted in patients with pulmonary artery hypertension (PAH), which indicates an important role for angiotensin and mineralocorticoid receptor antagonists in the treatment of PAH [22]. In Case 5, we detected high renin and aldosterone activity when she was noted to have biventricular hypertrophy. The combination of candesartan and captopril could improve her pulmonary arterial pressure, hypertrophic RV, and clinical condition. However, the pathophysiology of isolated high levels of renin and aldosterone was not clear in this case, and we speculate that she is in group 5 under the sixth WSPH classification system, with unclear or multifactorial mechanisms. The more specific categorization in the Panama system indicates group 6: a multifactorial pediatric pulmonary vascular hypertensive disease associated with other birth defects.

Regarding Case 6, Paolyta-B is an energy beverage from Paolyta Co., Ltd. (Taipei, Taiwan). It contains extracts of angelica, ginseng, and chuanxiong, amino acids, vitamins, and taurine. Commercially, it is claimed to refresh and provide nutrition to improve health. However, the safety of its use in pregnancy is unknown. Its high taurine content (1.0 mg/mL) caused us to explore the relationship between taurine and prenatal ductal arteriosus closures. Several studies have shown that taurine chloramine can inhibit the production of nitric oxide and prostaglandin E2 by suppressing inducible nitric oxide synthase and cyclooxygenase-2 expressions in various cells [26,27]. However, there is no direct evidence that taurine consumption can induce prenatal ductus arteriosus closure. We propose a molecular mechanism in which taurine causes the inhibition of fetal cyclooxygenase and nitric oxide synthase, which reduces the production of nitric oxide and prostaglandin E2, thus leading to prenatal ductal arteriosus closure. We recommend that pregnant women only ingest dietary supplements containing taurine if their metabolism and placental transport have been examined. There is no specific group for taurine-related PH in the sixth WSPH system, although Panama classification gives this case as group 1: prenatal or developmental pulmonary vascular hypertensive disease.

Regarding Case 7, SJMS (first reported in 1953) is a rare cause of unilateral hyperlucent hemithorax. This is a relatively uncommon entity, occurring in 3.8% to 4.3% of patients with bronchiolitis obliterans [28]. Most patients are asymptomatic and some, such as our case, present with repeated pneumonia, dyspnea, or hemoptysis [28]. The characteristic radiographic findings include a unilateral hyperlucent lung and can be diagnosed via echocardiography, chest computed tomography, selective pulmonary angiogram, flexible bronchoscopy, and pulmonary V/Q scans [29]. In a literature review, we found only a few case reports mentioning an association between SJMS and PH, and most of these occurred in adulthood [30,31,32,33,34]. Under the sixth WSPH classification, SJMS is group 5, PH with unclear or multifactorial mechanisms, and it can be categorized as group 5 in the Panama classification, isolated pediatric pulmonary hypertensive vascular disease, and group 3 in the simplified classification, developmental problems.

In conclusion, we emphasize perinatal pulmonary vascular maladaptation, maldevelopment, and pulmonary hypoplasia as causative factors in pediatric PH. The genetic, chromosomal, and multiple congenital malformation syndromes in the presentation of pediatric PH are very different from those in adults. There are some doubtfully classified and unclassified cases when applying the sixth WSPH system to pediatric PH, whereas the Panama system seems to provide a more practicable classification in pediatric PH. Further advances in molecular techniques are desirable for diagnosing the forms of PH uniquely found in children.

## Figures and Tables

**Table 1:** Clinical and imaging characteristics of the patients with unique PH.

Case No.	Age/Sex	Special Consideration	Classification			SPAP (mmHg)	Major Medical Treatment to PH	Prognosis
			6th WSPH [1]	Panama [4]	Simplified [2]			
1	1 d/M	47XXY	1	IV	I	70	PGE1	Expired (1 d)
2	52 d/M	Idiopathic	1	II	I	84	F + D + S + iNO + iPGI_2_	Expired (3 mo)
3	1 y/M	BPD	3	IV	I	93	F + S + iPGI_2_	Expired (3 y)
4	1 y/F	BPD	3	IV	I	66	F + S + iNO	Stable
5	1 d/F	High renin and aldosterone	5	VI	I	95	S + F + iNO + ARB + ACEI	Expired (7 y)
6	1 d/F	Taurine	unclear	I	I	55	F+iNO	Stable
7	3 y/M	Swyer–James syndrome	5	V	III	59	F + D + S + ACEI	Expired (4 y)

6th WSPH: 6th World Symposium on Pulmonary Hypertension; SPAP, systolic pulmonary artery pressure measured by cardioechography; d, days; y, years; BPD, bronchopulmonary dysplasia; F, furosemide; S, sildenafil; D, digoxin; iPGI_2_, inhaled prostacyclin (iloprost); iNO: inhaled nitric oxide; ARB, angiotensin receptor blocker; ACEI, angiotensin-converting enzyme inhibitor.
